# Predictive Modeling for the Growth of *Salmonella* spp. in Liquid Egg White and Application of Scenario-Based Risk Estimation

**DOI:** 10.3390/microorganisms9030486

**Published:** 2021-02-25

**Authors:** Mi Seon Kang, Jin Hwa Park, Hyun Jung Kim

**Affiliations:** 1 Korea Food Research Institute, Wanju, Jeollabuk-do 55365, Korea; 50035@kfri.re.kr (M.S.K.); parkjinhwa@kfri.re.kr (J.H.P.); 2 Department of Food Biotechnology, University of Science and Technology, Daejeon 34113, Korea

**Keywords:** *Salmonella* spp., liquid egg white, predictive model, validation, probability of infection

## Abstract

The objective of the study was to develop a predictive model of *Salmonella* spp. growth in pasteurized liquid egg white (LEW) and to estimate the salmonellosis risk using the baseline model and scenario analysis. Samples were inoculated with six strains of *Salmonella*, and bacterial growth was observed during storage at 10–37 °C. The primary models were developed using the Baranyi model for LEW. For the secondary models, the obtained specific growth rate (μ_max_) and lag phase duration were fitted to a square root model and Davey model, respectively, as functions of temperature (R^2^ ≥ 0.98). For μ_max_, the values were satisfied within an acceptable range (A*_f_*, B*_f_*: 0.70–1.15). The probability of infection (P*_inf_*) due to the consumption of LEW was zero in the baseline model. However, scenario analysis suggested possible salmonellosis for the consumption of LEW. Because *Salmonella* spp. proliferated much faster in LEW than in egg white (EW) during storage at 20 and 30 °C (*p* < 0.01), greater P*_inf_* may be obtained for LEW when these products are stored at the same conditions. The developed predictive model can be applied to the risk management of *Salmonella* spp. along the food chain, including during product storage and distribution.

## 1. Introduction

Eggs are a globally popular foodstuff due to their nutritious value and use as a material in other foods. They can be consumed in various forms: whole eggs, egg yolk, and egg white in food industries. Liquid egg products including all three forms are used widely in bakery industries and institutional food service systems where mass cooking or production takes place [1].

Even though eggs have natural defenses to prevent contamination by microorganisms, they are one of the main sources of *Salmonella* Enteritidis infections in humans [2]. The eggshell is a physical barrier that protects the inside of an egg and is mainly composed of calcium carbonate. However, due to the permeability of the eggshell membrane, it is possible that bacteria can enter eggs through the pores. As a chemical barrier, egg white (egg albumen) is generally a hostile environment for bacterial survival and growth because of its inherent antimicrobial proteins, such as lysozyme, conalbumin (ovotransferrin), ovomucin, and avidin [2].

Despite these antimicrobial characteristics, consumers have been concerned with a potential risk of foodborne disease occurring from the intake of eggs contaminated with *Salmonella* spp. Majowicz et al. (2010) [3] reported that 93.8 million cases of gastroenteritis are estimated per year globally due to nontyphoidal *Salmonella* infection, accompanied by 155,000 deaths. This indicates that salmonellosis is a considerable problem in developed countries as well as developing countries. In Korea, a country-wide *Salmonella* outbreak occurred, with 2207 cases in 2018. Epidemiological investigation revealed that the sources of infection were chocolate cakes that had been provided as school meals. In detail, the egg white used for making the cream in the cake was infected with *Salmonella* Thompson [4]. Therefore, studies on the behavior of *Salmonella* spp. in the different types of egg products are needed.

Liquid egg products are classified into pasteurized and unpasteurized types after processing. In the process of breaking the eggshells, liquid eggs are vulnerable to contamination with pathogenic bacteria. Even if a sterilization process is conducted, some bacteria can remain because pasteurization is carried out at 55 °C for liquid egg white to avoid the denaturation of the proteins in the egg white [5]. The Animal and Plant Quarantine Agency (APQA) in Korea reported that *Salmonella* Bareilly was detected in pasteurized liquid eggs, and the percentage of *Salmonella* positive eggs was 4.17% (5/120) [6]. In the United States, the USDA Food Safety and Inspection Service (USDA FSIS) began testing pasteurized egg products for *Salmonella* in 1995. In the testing program, the percentage of *Salmonella*-positive pasteurized liquid egg whites from 2008 to 2017 was 0.28% (9/3219) [7].

*Salmonella* spp. in egg whites can survive and grow under a favorable temperature range. Several studies have reported that *Salmonella* spp. are able to grow in separated fresh egg white at room temperature [8,9,10]. Because possible differences in the growth kinetics of *S.* Typhimurium and *S.* Sofia on eggs were reported [11], predictive modeling with cocktails of *Salmonella* spp. can provide more information on the behavior of *Salmonella* spp. in food. The use of diverse strain composites has been recommended for food safety studies such as predictive modeling because the variation of intra-species growth behavior may have a crucial effect on the quantitative microbial risk assessment [12]. For pasteurized liquid egg white (LEW), Huang (2015) [13] and Huang and Hwang (2017) [14] conducted the predictive modeling of a single strain, *Salmonella* Enteritidis. Kim et al. (2018) [9] reported that growth of *Salmonella* spp. consisting of five *Salmonella* serovars (*S.* Bareilly, *S.* Richmond, *S.* Typhimurium monophasic, *S*. Enteritidis, and *S*. Gallinarum) was modeled in egg products, but little information was available for the predictive growth model of different *Salmonella* serovars in pasteurized LEW.

Quantitative microbial risk assessment is a tool for evaluating the risk of infection when exposed to microorganisms and can assist in managing microbial food safety hazards and minimizing foodborne outbreaks [15]. Using the growth kinetic parameters as an essential element of quantitative microbial risk assessment leads to a more realistic picture of the risk management options available to control the risk of *Salmonella* spp. from contaminated food [16].

In this study, we developed a predictive model for the growth of *Salmonella* spp. in pasteurized LEW. We developed the primary and secondary model for the growth of four *Salmonella* serovars (*S.* Typhimurium, *S.* Enteritidis, *S.* Montevideo, and *S.* Kentucky) in pasteurized LEW and compared them to the primary model developed for fresh EW at a temperature favoring noticeable *Salmonella* spp. growth.

With the predictive model established in this study, we developed a probabilistic risk model describing the fate of LEW products as well as the growth of *Salmonella* spp. in LEW during the food chain and estimated the probability of illness from the consumption of LEW products adopting baseline model and scenario for the initial contamination level of *Salmonella* spp. in LEW. This study could provide useful information for conducting risk assessments of *Salmonella* spp. for egg white products.

## 2. Materials and Methods

### 2.1. Preparation of Egg Samples and Bacterial Cultures

Fresh shell eggs were purchased from a local market located in Jeonju, South Korea, and pasteurized LEW was purchased from an online retail market in South Korea. Fresh shell eggs were separated into egg yolk and egg white (EW) in a sterile biosafety cabinet (Esco Micro Pte. Ltd, Singapore, Singapore). The collected EW (stored in sterile sampling bags) (Merck, Darmstadt, Germany) were homogenized by a stomacher (BagMixer, Interscience, St. Nom, France) to ensure the consistency of the samples. Homogenized EW were subdivided into 10 mL aliquots in sterile conical tubes (SPL, Pocheon, Korea), and pasteurized LEW were also prepared in the same way. The sample tubes were prepared to fulfill the number of data points for each temperature.

To determine the mathematical models of *Salmonella* spp. in EW and LEW, six strains of *Salmonella* serovars were used in the experiments. *S*. Enteritidis (ATCC 13076, NCCP 14546), *S*. Typhimurium (NCCP 16207, NCCP 12219), *S*. Montevideo (NCCP 10140), and *S*. Kentucky (NCCP 11686) were provided by the American Type Culture Collection (ATCC) and the National Culture Collection for Pathogens (NCCP). All strains were individually inoculated with 10 mL Tryptic Soy Broth (Merck, Darmstadt, Germany), cultured at 37 °C, and shaken (140 rpm) overnight. The cultures were combined to make a cocktail of bacteria, which was used to inoculate the samples.

### 2.2. Inoculation and Measurement of Bacterial Counts

All samples (10 mL) were inoculated with 0.1 mL *Salmonella* spp. cocktail to obtain an initial density of 3.5 ± 0.5 log CFU/mL. The inoculated samples were stored at 5, 10, 15, 20, 25, 30, and 37 °C under isothermal conditions. To measure bacterial cell counts, each sample was taken at different time intervals for each temperature, and 10 mL sterilized saline was added to each sample and homogenized via vortexer. Ten-fold dilutions of samples were plated in duplicate onto Xylose Lysine Deoxycholate Agar (XLD, Oxoid, Basingstoke, UK), and the agar plates were incubated at 37 °C for 24 h. Typical colonies were counted and converted to log CFU/mL.

### 2.3. Development of Mathematical Models

The development of various mathematical models was carried out with the development of the primary model for each temperature and the secondary model for the parameters from the primary model.

The primary model for LEW was developed based on the Baranyi model [17], using the Microsoft Excel add-in DMFit version 3.5 (IFR, Norwich, UK).

(1) $$N_{t}=N_{0}+\mu_{\max}\times A_{t}-\ln\left[1+\frac{\exp\left(\mu_{\max}\times A_{t}\right)-1}{\exp\left(N_{\max}-N_{0}\right)}\right]$$

(2) $$\mathrm{where}\;A_{t}=t+\frac{1}{\mu_{\max}}\ln\left(\frac{\exp\left(-\mu_{\max}\times t\right)+h_{0}}{1+h_{0}}\right)$$

where A_t_ is the adjustment function, μ_max_ is the maximum specific growth rate, N_0_ is the initial bacterial cell count, N_max_ is the final bacterial count; h_0_ is a parameter defining the initial physiological state of the cells, and t is time.

The goodness of fit for the data was assessed by the coefficient of determination (R^2^), which was also provided by DMFit.

Because the growth curves for EW didn’t show a typical sigmoidal curve, the modeling was not conducted except for two temperatures. The growth curves at 20 and 30 °C were suitable to develop the primary model, but they only had exponential and stationary phases without showing distinct lag phases. Therefore, in case of EW, a three-parameter logistic model was used [18] with Graph Pad Prism version 5.0 (GraphPad Software, San Diego, CA, USA).

(3) $$N_{t}=N_{0}+N_{\max}-\ln[\exp\left(N_{0}\right)+\left(\exp\left(N_{\max}\right)-\exp\left(N_{0}\right)\right)\times \exp\left(-\mu_{\max}\times t\right)$$

The specific growth rate (μ_max_) estimated from the primary models of EW was used to compare the growth of *Salmonella* spp. in EW and LEW.

From the primary models describing the growth of *Salmonella* spp. in pasteurized LEW, the estimated lag phase duration (LPD) and maximum specific growth rate (μ_max_) were used to develop the secondary model. The Davey model [19] and square root model [20] were used to fit LPD and μ_max_, respectively, with Graph Pad Prism version 5.0.

Davey model: LPD = a + (b/T) + (c/T^2^) (4)

where a, b, and c are regression coefficients, and T is temperature.

Square root model: μ_max_ = [a(T − T_min_)]^2^ (5)

where a is the slopes of the regression lines for μ_max_, T is temperature, and Tmin is the theoretical minimum temperature for growth.

### 2.4. Model Validation

To validate the developed models, pasteurized LEW samples were stored at 20 and 30 °C, which were not used in the primary models. The obtained data from the experiments and the predicted data from the developed models were assessed using the accuracy factor (A*_f_*), bias factor (B*_f_*) [21], and root mean square error (RMSE) [22]. When the observed values and the predicted values match exactly, A*_f_* = B*_f_* = 1. An RMSE value close to zero indicates that the data closely fit the model.

A*_f_*= 10 ^(∑|log(predicted/observed)|/n)^ (6)

B*_f_*= 10 ^(∑log(predicted/observed)/n)^ (7)

(8) $$\mathrm{RMSE}=\;\sqrt{\;\frac{1}{n}\sum^{\;}{\left(\mathrm{predicted}-\mathrm{observed}\right)}^{2}}$$

### 2.5. Probabilistic Risk Modeling and Scenario Analysis

To estimate the risk of *Salmonella* spp. from the consumption of LEW, we considered the distribution of LEW products from the manufacturers to institutional food service facilities, where they are cooked and served to consumers. Logical schemes for the probabilistic risk model are shown in Figure S1. A simulation model was established, with four steps consisting of the initial contamination level of *Salmonella* spp. in LEW, the transportation and storage conditions (time and temperature) of the manufacturer and institutional food service system, and the daily consumption amounts. The developed model, combined with the time and temperature information, was used to estimate the bacterial growth in LEW from the manufacturer site to consumption. We adopted a current standard of *Salmonella* spp. in LEW provided by the Korea Food Code [5] as a baseline model, then applied the scenario of the initial contamination data in the LEW to identify the possible changes in the probability of infection with different initial contamination levels and to suggest risk management options. With the developed probabilistic risk model, we conducted Monte Carlo simulations to estimate the probability of infection. The simulation was performed with 100,000 iterations using @RISK software version 7.6 (Palisade Corporation, Ithaca, NY, USA).

The scenario analysis used for LEW could not applied to EW due to its insufficient mathematical model. Therefore, a simple scenario was designed for EW, describing the situation wherein EW with a certain initial contamination level was exposed to a specific time at isothermal conditions (20 or 30 °C). This scenario was also applied to the LEW under the same time and temperature conditions for comparing the risk of infection.

### 2.6. Statistical Analysis

All the data were obtained from at least three independent experiments with two replicates, with nine experiments as the maximum. A significant difference (*p* < 0.01) in μ_max_ between EW and LEW was analyzed via *t*-test with IBM SPSS Statistics version 20 software.

## 3. Results and Discussion

### 3.1. Development of a Predictive Model for LEW

**Figure 1:**
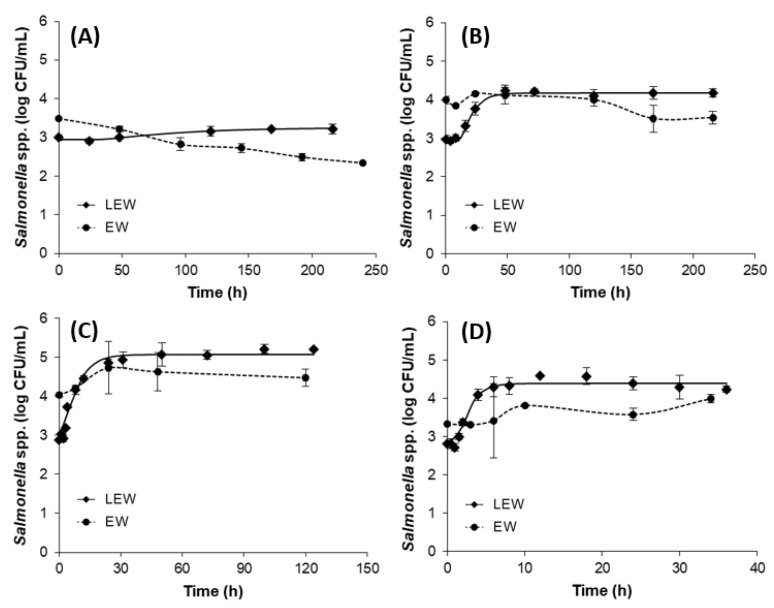
Primary model of the growth of *Salmonella* spp. in liquid egg whites (LEW) and growth curve in egg whites (EW). (**A**) 10 °C; (**B**) 15 °C; (**C**) 25 °C; (**D**) 37 °C. ♦: observed value for LEW, solid line: fitted line of primary model for LEW, ●: observed value for EW, dashed line: growth or survival curve for EW.

No growth of *Salmonella* spp. was observed at 5 °C both in EW and in LEW. When the samples were stored at 10 °C, *Salmonella* spp. did not proliferate in EW but showed a slight proliferation in LEW. At a temperature above 15 °C, the growth of *Salmonella* spp. was observed in LEW, but the maximum population density was less than 6 log CFU/mL (Figure 1), whereas *Salmonella* spp. grew up to 9 log CFU/mL in egg yolk [9,23]. Growth of *Salmonella* spp. in EW was not observed at 15 °C. It is well known that egg white is a generally inadequate environment for the survival and growth of bacteria due to its natural antimicrobial barriers (such as lysozyme and ovotransferrin), but some kinds of *Salmonella* serovars can proliferate in nonrefrigerated egg white, and the bacteria transfer to nutritious egg yolk and multiply to a high density [2]. A recent study suggested that the natural concentration of the extra fatty acid binding protein (Ex-FABP) in egg white appears sufficient to play a biological role in limiting bacterial growth through the sequestration of the siderophore enterobactin. However, this effect is overcome by *S.* Enteritidis through its ability to deploy a salmochelin as a second, “stealth” siderophore [24].

**Figure 2:**
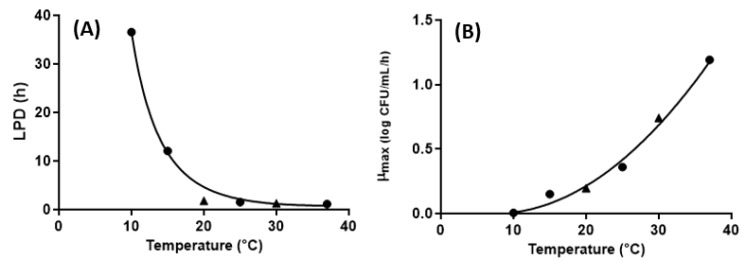
Secondary model and validation at 20 and 30 °C for *Salmonella* spp. growth in LEW. (**A**) lag phase duration (LPD); (**B**) μ_max_. ●: observed value for secondary model, solid line: fitted line of secondary model, ▲: observed value for validation.

The primary models were developed using the Baranyi model for LEW. The kinetic parameters determined by primary models showed that, as temperature increased, μ_max_ increased from 0.006 to 1.192 and LPD values decreased from 36.571 to 1.157 (Table 1). Then, the secondary models were developed using the square root model and Davey model for μ_max_ and LPD, respectively, finding that the goodness of fit was above 0.98 for both secondary models. The secondary model showed positive correlation for μ_max_ and negative correlation for LPD as temperature increased (Table 2 and Figure 2). The μ_max_ estimated from the secondary model reported by Huang (2015) [13] ranged from 0.081 at 10 °C to 0.921 at 37 °C and was developed for *S*. Enteritidis in pasteurized LEW. Generally, the μ_max_ values provided by the previous report [13] were higher than those obtained in this study. Additionally, *S*. Enteritidis and *S*. Typhimurium only grew slightly at 25 °C and did not proliferate at 8, 10, 15, and 35 °C in egg white [10], and *Salmonella* spp. consisting of five serovars maintained the initial populations at 25 and 30 °C in unpasteurized liquid egg white [9]. These growth patterns made it difficult to develop the mathematical model for EW and unpasteurized LEW. Similar results were observed in this study, meaning that the primary model could be developed for some temperatures, but the secondary model could not be used for fresh EW in this study.

**Figure 3:**
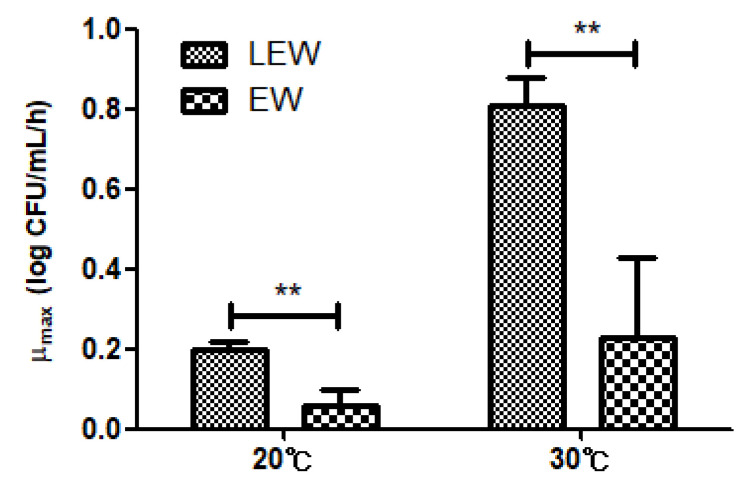
Specific growth rate for *Salmonella* spp. in LEW and EW stored at 20 and 30 ℃. ** A significant difference between EW and LEW (*p* < 0.01, *t*-test with IBM S*PSS* Statistics version 20).

To compare the growth of *Salmonella* spp. in EW and LEW, the growth data for two selected temperatures were fitted using the Baranyi model for LEW and the three-parameter logistic model for EW. The growth curves for LEW generally exhibited three distinct growth phases (the lag, exponential, and stationary phases) but were not observed for EW. Since the growth curves in EW did not show noticeable lag phases, only the specific growth rate was compared. During storage at 20 and 30 °C, the specific growth rate was much higher in LEW than in EW (*p* < 0.01; Figure 3). This result represented that the risk of foodborne illness is potentially higher for consumption of LEW contaminated with *Salmonella* spp. than EW.

### 3.2. Validation of the Predictive Model

The secondary models for the growth of *Salmonella* spp. in LEW were validated based on the growth data of *Salmonella* spp. in LEW at 20 and 30 °C that had not been used to develop the primary model (Table 2 and Figure 2). To determine the performance of the mathematical models, the observed values (μ_max_ and LPD) at 20 and 30 °C were compared with the predicted values from the developed secondary models. The deviation between the observed and predicted values was evaluated by three indices: bias factor (B*_f_*), accuracy factor (A*_f_*), and root mean square error (RMSE). The bias factor describes whether the developed model is over or under prediction. It is generally interpreted that a B*_f_* value of 0.9–1.05 is good, while values of 0.7–0.9 and 1.06–1.15 are acceptable ranges for predictive models [25]. A successful validation between predictions and observation results is indicated by bias and accuracy factors equal to one and RMSE values close to zero.

For μ_max_, the B*_f_*, A*_f_*, and RMSE of the secondary models were 1.003, 1.084, and 0.041, respectively. All values were within the acceptable range, so the models could be used to predict the specific growth rate of *Salmonella* spp. in pasteurized LEW. In contrast, for LPD, the three indices were calculated as 1.546, 1.650, and 2.004, respectively. It was interpreted from the validation results that the developed model for LPD could not describe the lag time successfully. This was due to *Salmonella* spp. having a relatively long lag time at 10 °C (36.57 h) and 15 °C (12.09 h), but the lag time decreased sharply above 15 °C, and there was a little difference from 20 °C (1.83 h) to 37 °C (1.16 h).

The predictive models for LEW were developed using a no lag phase model, and only the specific growth rate was reported [13,14]. Even though the secondary model of LPD was assessed to a low level of confidence in this study, we experimentally determined that *Salmonella* spp. had a noticeable lag phase duration. In this regard, the model developed here might provide useful information for risk management in LEW contaminated with *Salmonella* spp.

### 3.3. Probabilistic Risk Modeling and Scenario Analysis

To estimate the probability of infection (P*_inf_*), we established a scenario that comprised of four steps (Figure S1). In establishing the scenario model, we assumed that (1) people consume food containing undercooked or unheated LEW, and (2) the contamination level of *Salmonella* spp. in LEW was at the same level as that from the literature. In order to develop the baseline model, we assumed that the LEW products were manufactured in accordance with the guidelines of the Korean Food Code [5], i.e., the initial contamination of *Salmonella* spp. LEW was assumed as zero for 25 g of sample. A different scenario was applied to the initial contamination level, i.e., data on the prevalence of *Salmonella* spp. in LEW from the literature were applied to determine the possible risk from the LEW consumption. The prevalence data from 2008 to 2017 for *Salmonella* spp. in LEW reported by the USDA FSIS (2018) [7] in America were used as the initial contamination level for scenario 1. Since 1995, the USDA FSIS has been conducting a monitoring program for pasteurized egg products, which includes the mandatory inspection of processed egg products. In Korea, the APQA (2011) [6] reported the prevalence of *Salmonella* spp. in pasteurized egg products from eight manufacturers, and this report was the only published domestic data source to the best of our knowledge. Therefore, we adopted the prevalence data in Korea as the initial contamination level for scenario 2.

After determining the initial contamination level, the growth of *Salmonella* spp. was estimated by the mathematical models developed in this study according to the distribution of time and temperature that the products might be exposed to during transportation, storage, and food serving. The time and temperature distribution data during manufacturing were obtained through personal communication. For institutional food service, the time and temperature data during storage and food serving were obtained from the previous work [26]. The same data on the distribution of time and temperature were used for both the baseline model and the scenario analyses on the initial contaminations. In the final step, the P*_inf_* was estimated using the dose–response model, combined with the consumption amount of LEW and the estimated contamination of *Salmonella* spp. in LEW at the consumption level. The used dose–response model was as follows: Beta-Poisson model: P_ill_ = 1 − (1 + dose/2885)^−0.3126^. This model was derived from nontyphi *Salmonella* human feeding trial data, covering all *Salmonella* serotypes [27]. The daily consumption of egg white products was calculated based on the data from the Korea National Health and Nutrition Examination Survey [28] (Table S1).

In this risk model, the LPD values were used to determine the initial physiological state of the cell (the h_o_ value) with the μ_max_. There was little difference between predicted LPD (1.19 h) and observed LPD (1.27 h) at 30 °C, whereas the predicted LPD was 4.66 h and the observed LPD was 1.83 h at 20 °C, and this deviation led to the lower level of confidence for the secondary model. However, the h_o_ values were similar for both prediction (h_o_ = 0.932) and observation (h_o_ = 0.991) because they were calculated as the average of the LPD multiplied by the μ_max_. Since this deviation of h_o_ does not notably affect the risk estimation, we used the developed model for LEW in the scenario analysis.

The simulation results of the baseline model showed that the P*_inf_* was estimated to be zero, since food standards in Korea do not allow *Salmonella* spp. to be detected in egg products [5]. In June 2020, MFDS reported through a press release that foodborne pathogenic bacteria including *Salmonella* were not detected in 241 liquid egg products from 160 domestic manufacturers. According to the scenario of the initial contamination level (i.e., 3.11 × 10^−5^ CFU/g and 2.00 × 10^−3^ CFU/g for scenarios 1 and 2, respectively), the estimated mean values of the P*_inf_* (per person per day) were 2.03 × 10^−7^ and 1.32 × 10^−5^ for scenarios 1 and 2, respectively, as estimated from the 100,000 iterations (Table 3). We also conducted a sensitivity analysis based on the regression coefficients for the variables used for the simulation model. The results showed that the daily consumption of egg white products was a predominant factor affecting the occurrence of foodborne illnesses, and the initial contamination level was the second factor for both scenarios 1 and scenario 2.

Based on the result that the specific growth rate of *Salmonella* spp. was greater in LEW than in EW, we simulated a simple scenario model to compare the P*_inf_* from the consumption of two types of egg white. In this scenario, we used the initial contamination level obtained from USDA FSIS (2008–2017) [7] as used in scenario 1, but the time and temperature distributions used in the scenario model were not applied to this analysis, since the primary model could only be fitted at 20 and 30 °C for EW. Therefore, we simply compared the P*_inf_*, assuming that the LEW and EW contaminated with *Salmonella* spp. are exposed for a certain time at specific temperature conditions (20 and 30 °C; Tables S2 and S3). Table 4 shows the mean of P*_inf_* per person per day in LEW and EW at the specified temperatures and time durations. Generally, the P*_inf_* increased as the exposure temperature and time increased for both LEW and EW. When comparing LEW and EW, the P*_inf_* was higher in LEW than in EW under every condition. When the egg white products were exposed for 12 h at 20 °C and 4 h at 30 °C, the estimated P*_inf_* of the LEW was 3.8-fold and 65-fold higher than those of EW products, respectively. When exposure time increased, the estimated fold changes of P*_inf_* (LEW) and P*_inf_* (EW) increased dramatically. As mentioned, in 2018, an egg-associated salmonellosis outbreak that occurred in Korea resulted in about 2000 cases due to chocolate cake provided as a school meal service. Egg white contaminated with *Salmonella* Thompson was identified as the cause of infection, used as a raw material for the cream in the chocolate cake [4]. In this institutional food service (e.g., a school meal system), liquid egg products, rather than shell eggs, are mainly distributed, due to their convenience in mass cooking. However, most previous studies involving the development of a predictive model and risk assessment have been developed considering whole eggs or egg yolk from both shell eggs and liquid eggs except for egg white [9,23,29,30,31]. There have been a few studies on LEW, but only one serovar (*S*. Enteritidis) was used to develop the model [13,14]. In this study, we used a mixture of four *Salmonella* serovars, considering the variation of intraspecies growth behavior. Furthermore, a comparative study was carried out for LEW and EW to show the need for the risk management of egg white products to prevent possible foodborne disease. Therefore, the developed models for LEW and the comparative study on LEW and EW here can be useful information for quantitative microbial risk assessment of *Salmonella* spp. during consumption of foods associated with egg white products.

However, the developed probabilistic risk model has some limitations. Firstly, we adopted contamination of *Salmonella* spp. in LEW from the literature and used as initial contamination level in the scenario analysis; one scenario used data from the United States, and the other utilized Korean prevalence data from 2011. In order to obtain a clearer picture of salmonellosis due to LEW or LEW containing foods, further study on the prevalence of *Salmonella* spp. in LEW is needed. Secondly, we assumed that LEW was consumed raw due to lack of compliance data on the handling of eggs, especially LEW; thus, the obtained P*_inf_* as a result of simulation may be overestimated. In order to improve the risk assessment result, information is needed on the consumption of food containing raw or undercooked EW; for example, noncompliance with the guidelines of time and temperature for food handling at food service establishments or consumption data for food containing raw LEW.

## 4. Conclusions

For the primary model, the growth of *Salmonella* spp. in liquid egg white (LEW) was fitted to the Baranyi model, and the obtained LPD and μ_max_ values were then fitted as a function of temperature using the Davey model and square root model. On the contrary, the growth of *Salmonella* spp. in fresh egg white (EW) could be fitted only at 20 and 30 °C for the primary model, so the development of the secondary model was not carried out. Comparing the growth of *Salmonella* spp. in LEW as well as EW, it was found that the growth of *Salmonella* spp. in LEW is faster than in EW (*p* < 0.01). We estimated the probability of infection using Monte Carlo simulations, adopting a baseline model based on the current practices of the distribution and consumption of LEW and a scenario analysis for liquid egg white with a possible initial contamination level obtained from the literature. The risk of salmonellosis due to the consumption of LEW was zero in the baseline model. However, scenario analysis suggested that the mean P*_inf_* was 2.03 × 10^−7^ (scenario 1) and 1.32 × 10^−5^ (scenario 2) per person per day, according to currently available data. In addition, we showed the differences in P*_inf_* from the consumption of LEW and EW during storage (or distribution) at 20 and 30 °C. The faster growth of *Salmonella* spp. in LEW than in EW indicates that greater P*_inf_* may be obtained for LEW when these products are stored at the same temperature and for the same length of time.

The developed predictive model in this study can be applied to estimate the probability of foodborne disease associated with *Salmonella* spp. growth along the food chain, including in product storage and distribution. Consequently, LEW, which is mainly used for mass cooking in the food service industry, showed stable and predictable proliferation of *Salmonella* spp. and was estimated to have a higher probability of risk than EW, suggesting that a strict standard is needed for safety management in processing liquid egg white.

## Figures and Tables

**Table 1:** Kinetic parameters as calculated by the Baranyi model for the growth of *Salmonella* spp. in LEW during storage at 10, 15, 25, and 37 °C.

Parameter	Temperature (°C)			
	10	15	25	37
μ_max_ (log CFU/mL/h)	0.006	0.150	0.361	1.192
LPD (h)	36.571	12.090	1.540	1.157
R^2^	0.876	0.992	0.977	0.976

**Table 2:** Secondary model of the growth of *Salmonella* spp. in LEW and validation of the developed model.

Parameter	Secondary Model		Validation		
	Equation	R^2^	B*_f_*	A*_f_*	RMSE
μ_max_	μ_max_ = (0.0366 × (T-7.359))^2^	0.990	1.003	1.084	0.041
LPD	5.048 + (−331.5/T) + (6475/T^2)	0.999	1.546	1.650	2.004

**Table 3:** Scenario analysis of *Salmonella* spp. exposure.

Scenario Analysis		Probability of Illness/Person/Day			
Simulation Model	Initial Contamination Level ^2^	5%	Mean	95%	99%
Baseline model ^1^	0/25 cell/g	0	0	0	0
Scenario 1	3.11 × 10^−5^ CFU/g	1.38 × 10^−8^	2.03 × 10^−7^	5.24 × 10^−7^	7.12 × 10^−7^
Scenario 2	2.00 × 10^−3^ CFU/g	8.37 × 10^−7^	1.32 × 10^−5^	3.61 × 10^−5^	5.16 × 10^−5^

^1^ Baseline model: Simulation model under the current standard according to Korean Food Code [5]. ^2^ Initial contamination level for the scenario model in this table is the mean value of distribution from prevalence data using @RISK in Table S1.

**Table 4:** Comparison of the probabilities of risk in LEW and EW at 20 and 30 °C.

Exposure Temperature (°C)	Exposure Time (h).	Mean of Probability of Infection/Person/Day	
		EW	LEW
20	12	9.83 × 10^−7^	3.69 × 10^−6^
	24	5.75 × 10^−6^	5.98 × 10^−4^
	36	3.37 × 10^−5^	9.61 × 10^−2^
30	4	1.37 × 10^−6^	8.91 × 10^−5^
	8	1.12 × 10^−5^	9.54 × 10^−2^
	12	9.07 × 10^−5^	7.57 × 10^−1^

## Data Availability

Not applicable.

## Supplementary Materials

The following are available online at https://www.mdpi.com/2076-2607/9/3/486/s1, Figure S1: Flow chart for estimation of the probability of infection for *Salmonella* spp. in LEW, Table S1: Simulation model and formula used to estimate the risk of *Salmonella* spp. in liquid egg white with @RISK, Table S2: Simulation model and formula used to estimate the risk of *Salmonella* spp. in liquid egg white at 20 and 30 °C with @RISK, Table S3: Simulation model and formula used to estimate the risk of *Salmonella* spp. in egg white at 20 and 30 °C with @RISK.
