# Supplementary material for: Predictive Modeling for the Growth of Salmonella spp. in Liquid Egg White and Application of Scenario-Based Risk Estimation
Source: Microorganisms. 2021 Feb 25;9(3):486. doi: 10.3390/microorganisms9030486 (PMC7996612; doi:10.3390/microorganisms9030486)
Supplement: Supplementary file 1 [file microorganisms-09-00486-s001.pdf]

microorganisms

Supplementary Materials: Predictive Modeling for the Growth of *Salmonella* spp. in Liquid Egg White and Application of Scenario-Based Risk Estimation

Mi Seon Kang[1,2], Jin Hwa Park [1] and Hyun Jung Kim [1,2,*]

Figure S1. Flow chart for estimation of the probability of infection for *Salmonella* spp. in LEW.

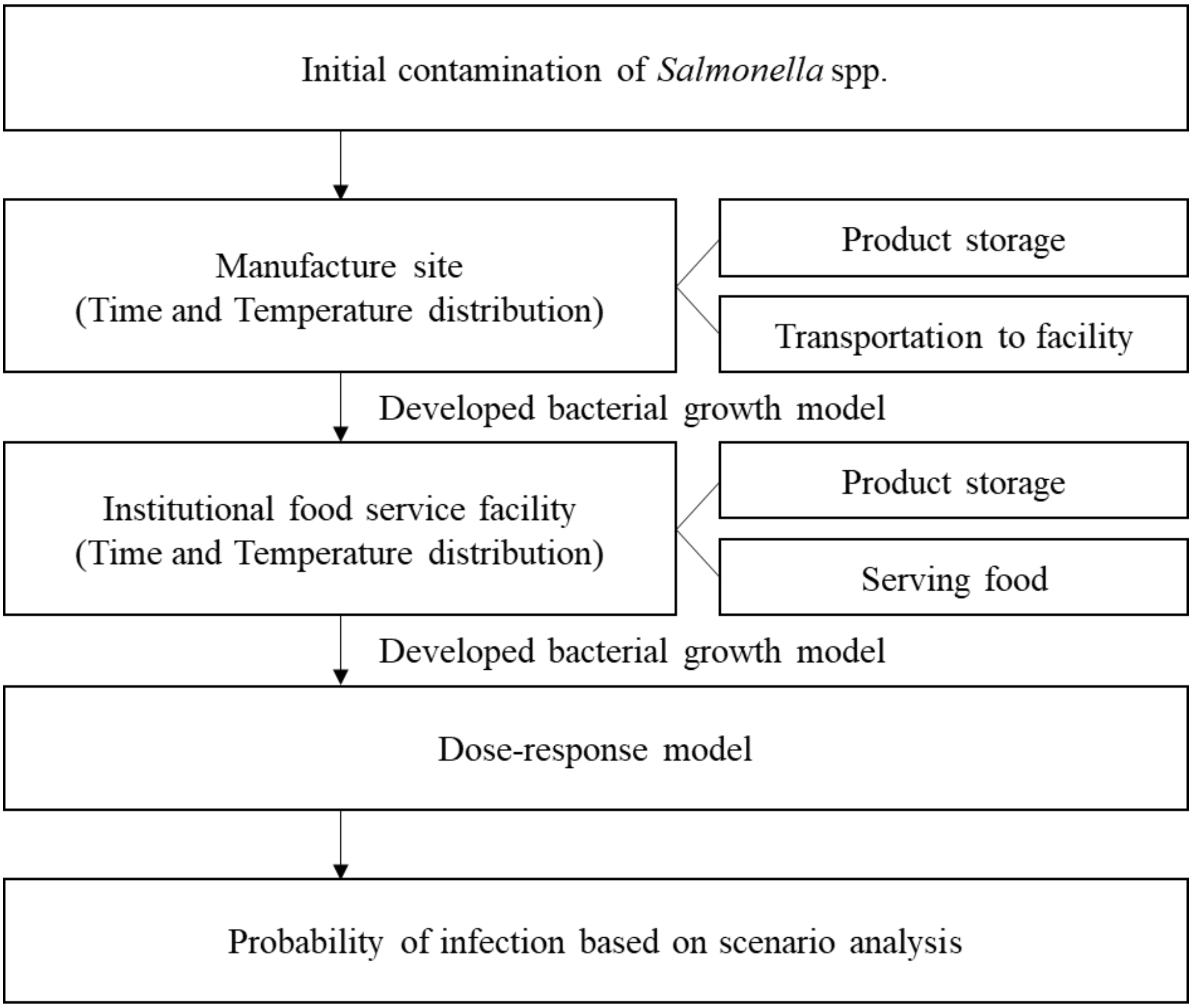

Table S1. Simulation model and formula used to estimate the risk of *Salmonella* spp. in liquid egg white with @RISK.

**Scheme 1.**	**Variable**	**Unit**	**Formula**	**Reference**
**Product**				
Initial contamination level of Scenario 1				
Prevalence of *Salmonella* spp.	PR		=Riskbeta(10,3211)	USDA FSIS (2018)
Initial contamination level	C	CFU/g	=-LN(1-PR)/100g	Haas et al (1999)
	IC	log CFU/g	=LOG(C)	
Initial contamination level of Scenario 2				
Prevalence of *Salmonella* spp.	PR		=Riskbeta(6,116)	APQA (2011)
Initial contamination level	C	CFU/g	=-LN(1-PR)/25g	Haas et al (1999)
	IC	log CFU/g	=LOG(C)	
**Storage in Manufacturer**				
Storage time	Manu-time	h	=RiskUniform(0,24)	Personal Communi-cation
Storage temperature	Manu-temp	℃	=RiskUniform(0,5)	Personal Communi-cation
Growth	h0		=average(growth rate*lag time), Fixed 0.991	*
	Y0		=average(Y0),Fixed2.918	*
	Yend		=average(Yend),Fixed4.226	*
	Manu-R	log CFU/g/h	=[0.0366(Manu-temp-7.359)]^2	*
	At		=Manu-time+1/Manu-R*ln((exp(-Manu-R*Manu-time)+h0)/(1+h0))	*
Salmonella spp. growth at manufacturer	Manu-G	log CFU/g	=IC+Manu-R*At-ln(1+((exp(Manu-R*At)-1)/(exp(Yend-IC))))	*
Salmonella spp. amount at manufacturer	C1	log CFU/g	=IF(Manu-temp>=10,Trans-G,IC)	*

(Continued)

Step	Variable	Unit	Formula	Reference
**Transportation (Manufacturer → Institutional food service)**				
Transportation time	Trans-time	h	=RiskUniform(0.5,12)	Personal Communication
Transportation temperature	Trans-temp	℃	=RiskUniform(0,5)	Personal Communication
Growth	h0		=average(growth rate*lag time), Fixed 0.991	*
	Y0		=average(Y0), Fixed2.918	*
	Yend		=average(Yend),Fixed4.226	*
	Trans-R	log CFU/g/h	=[0.0366(Trans-temp-7.359)]^2	*
	At		=Trans-time+1/Trans-R*ln((exp(-Trans-R*Trans-time)+h0)/(1+h0))	*
Salmonella spp. growth during transportation	Trans-G	log CFU/g	=C1+Trans-R*At-ln(1+((exp(Trans-R*At)-1)/(exp(Yend-C1))))	*
Salmonella spp. amount during transportation	C2	log CFU/g	=IF(Trans-temp>=10,Trans-G,C1)	*
**Storage in Institutional Food Service**				
Storage time	Inst-time	h	=RiskUniform(0.5,144)	Park (2020)
Storage temperature	Inst-temp	℃	=RiskUniform(3,7)	Park (2020)
Growth	h0		=average(growth rate*lag time), Fixed 0.991	*
	Y0		=average(Y0),Fixed2.918	*
	Yend		=average(Yend),Fixed4.226	*
	Inst-R	log CFU/g/h	=[0.0366(Inst-temp-7.359)]^2	*
	At		=Inst-time+1/Inst-R*ln((exp(-Inst-R*Inst-time)+h0)/(1+h0))	*
Salmonella spp. growth at institutional food service	Inst-G	log CFU/g	=C2+Inst-R*At-ln(1+((exp(Inst-R*At)-1)/(exp(Yend-C2))))	*
Salmonella spp. amount at institutional food service	C3	log CFU/g	=IF(Inst-temp>=10,Inst-G,C2)	*

(Continued)

Step	Variable	Unit	Formula	Reference
**Serving to consumers**				
Serving time	Serv-time	h	=Riskpert(0.17,1,2)	Park (2020)
Serving temperature	Serv-temp	℃	=RiskUniform(16,20)	Park (2020)
Growth	h0		=average(growth rate*lag time), Fixed 0.991	*
	Y0		=average(Y0),Fixed2.918	*
	Yend		=average(Yend),Fixed4.226	*
	Serv-R	log CFU/g/h	=[0.0366(Serv-temp-7.359)]^2	*
	At		=Serv-time+1/Serv-R*ln((exp(-Serv-R*Serv-time)+h0)/(1+h0))	*
Salmonella spp. growth during serving	Serv-G	log CFU/g	=C3+Serv-R*At-ln(1+((exp(Serv-R*At)-1)/(exp(Yend-C3))))	*
Salmonella spp. amount during serving	C4	log CFU/g	=IF(Serv-temp>=10,Serv-G,C3)	*
**Consumption**				
Daily Consumption	Consump	g	=RiskTriang(0.034615,0.034615,149.03)	KDCA (2018)
Salmonella spp. amount per daily consumption	D	CFU/ serving size	=10^C2*Consump	
**Risk**				
Probability of risk	risk		=1-[1+D/2885]^(-0.3126)	WHO (2002)

*: This research

Table S2. Simulation model and formula used to estimate the risk of *Salmonella* spp. in liquid egg white at 20 and 30 °C with @RISK.

Step	Variable	Unit	Formula	Reference
**Product**				
Prevalence of *Salmonella* spp.	PR		=Riskbeta(10,3211)	USDA FSIS (2018)
Initial contamination level	C	CFU/g	=-LN(1-PR)/100g	Haas et al (1999)
	IC	log CFU/g	=LOG(C)	
***Salmonella* growth at 20 °C**				
Time	Time	h	12, 24, 36	
Temperature	Temp	℃	20	
Growth at 20 ℃ (Baranyi model)	h0		0.394	*
	Y0	log CFU/g	2.98	*
	Yend	log CFU/g	4.81	*
	SGR	log CFU/g/h	0.2	*
	At		=Time+1/SGR*ln((exp(-SGR*Time)+h0) /(1+h0))	*
Salmonella spp. growth	Growth	log CFU/g	=IC+SGR*At-ln(1+((exp(SGR*At)-1) /(exp(Yend-IC))))	*
***Salmonella* growth at 30 °C**				
Time	Time	h	4, 8, 12	
Temperature	Temp	℃	30	
Growth at 30 ℃ (Baranyi model)	h0		1.411	*
	Y0	log CFU/g	2.74	*
	Yend	log CFU/g	4.71	*
	SGR	log CFU/g/h	0.809	*
	At		=Time+1/SGR*ln((exp(-SGR*Time)+h0) /(1+h0))	*
Salmonella spp. growth	Growth	log CFU/g	=IC+SGR*At-ln(1+((exp(SGR*At)-1) /(exp(Yend-IC))))	*

(Continued)

Step	Variable	Unit	Formula	Reference
**Consumption**				
Daily Consumption	Consump	g	=RiskTriang(0.034615,0.034615,149.03)	KDCA (2018)
Salmonella spp. amount per daily consumption	D	CFU/ consump	=10^C2*Consump	
**Risk**				
Probability of risk	risk		=1-[1+D/2885]^(-0.3126)	WHO (2002)

*: This research

Table S3. Simulation model and formula used to estimate the risk of *Salmonella* spp. in egg white at 20 and 30 °C with @RISK.

Step	Variable	Unit	Formula	Reference
**Product**				
Prevalence of *Salmonella* spp.	PR		=Riskbeta(10,3211)	USDA FSIS (2018)
Initial contamination level	C	CFU/g	=-LN(1-PR)/100g	Haas et al (1999)
	IC	log CFU/g	=LOG(C)	
***Salmonella* growth at 20 °C**				
Time	Time	h	12, 24, 36	
Temperature	Temp	℃	20	
Growth at 20 ℃ (Three parameter logistic model)	Y0	log CFU/g	3.91	*
	Yend	log CFU/g	4.98	*
	SGR	log CFU/g/h	0.064	*
Salmonella spp. growth	Growth	log CFU/g	=IC+Yend-LN(exp(IC)+(exp(Yend)-exp(IC))*exp(-SGR*Time))	*
***Salmonella* growth at 30 °C**				
Time	Time	h	4, 8, 12	
Temperature	Temp	℃	30	
Growth at 30 ℃ (Three parameter logistic model)	Y0	log CFU/g	3.5	
	Yend	log CFU/g	4.27	
	SGR	log CFU/g/h	0.228	
Salmonella spp. growth	Growth	log CFU/g	=IC+Yend-LN(exp(IC)+(exp(Yend)-exp(IC))*exp(-SGR*Time))	
**Consumption**				
Daily Consumption	Consump	g	=RiskTriang(0.034615,0.034615,149.03)	KDCA (2018)
Salmonella spp. amount per daily consumption	D	CFU/consump	=10^C2*Consump	
**Risk**				
Probability of risk	risk		=1-[1+D/2885]^(-0.3126)	WHO (2002)

*: This research
